# Hospital Expenditure.—The Commissariat

**Published:** 1896-07-04

**Authors:** 


### The Institutional Workshop.

### HOSPITAL EXPENDITURE.—THE COMMISSARIAT.

### XVIII.—PRICE OF MEAT IN THE PROVINCES.

The conditions of the meat supply are largely modified outside the metropolis. The contract system is not universal, many hospitals in country towns finding it better and cheaper to purchase in the open market. Moreover, it is by no means uncommon for the meat to be bought in the carcase. A lower price can, of course, always be quoted when this is done, and where suitable premises are available and the necessary skill for turning every part of the animal to good account there is a distinct saving, and a gain in variety. No London hospital could cope with an additional strain of this kind. The kitchen premises are not adapted for cutting up meat in bulk, nor are London cooks knowledgeable in the art of dealing economically with the inferior parts of the animal.

With the exception of this means of economy, and excluding a few provincial hospitals, mostly in large towns, which appear to contract for foreign meat, the prices rule distinctly higher in the country than in London. A few examples must suffice. The period covered when not otherwise mentioned is 1893.

At the Royal Infirmary, Newcastle-on-Tyne, the average of the various quarterly prices works out at 5½d. per lb. "Mutton is to consist of the whole sheep," and this no doubt accounts for the low price quoted for the whole.

Other hospitals quoting one price for the meat supply are:—

| | |
|---|---|
| Leeds General Infirmary ... ... | 5⅛d. per lb. all round. |
| Norfolk and Norwich ... ... ... | 6¼d. " " " |
| Wolverhampton and Staffordshire ... | 6¼d. " " " |
| Sheffield General Infirmary ... ... | 6½d. " " " |
| York County ... ... ... ... | 6¾d. (" tea-beef," 6d.) |
| Hull Royal Infirmary... ... ... | 7d. per lb. all round. |

The lowest price quoted is that of the Sussex County, where the prices vary from 4½d. to 4¾d., presumably for foreign meat.

At the Liverpool Royal Infirmary foreign meat is also used, the prices paid being 4¾d. and 5d. per lb.

In Birmingham, at both the Queen's and General Hospitals, the prices range higher. New Zealand mutton, 6½d. per lb., and other joints from 7¼d. to 8d.; shin, 4d.

At both Oxford and Cambridge the prices quoted are for 1894, they are from 7d. or 8d. per lb. for joints and 5½d. per lb. for shin, &c.

At Bristol the range is from 7d. for leg of mutton to 4¾d. for shin, and at the Derbyshire Royal Infirmary from 8d.

Enough has been said to show that the prices are, on the whole, higher than in London. The minimum of 2d. and 3½d. per lb., several times encountered among London prices, is nowhere touched; the lowest quotation is 4d. for shin of beef in Birmingham, and in no other place do prices sink below 4¾d., so far as returns have been received. And, again, the rate of 7d. to 8d. per lb., confined in London, as a rule, to the joints for the officers' table, is frequently given for the main supplies in the country.

In Scotch and Irish hospitals a further rise of prices is noticeable. With the exception of the Dundee Royal Infirmary and the Glasgow Royal Infirmary, where the meat is contracted for respectively at 6d. and 5½d. per lb. all round, the prices far exceed those given in London.

At the Edinburgh Royal Infirmary the prices are 8d. and 6¾d., with 5d. per lb. for forequarters of beef.

At the Aberdeen Royal Infirmary 8½d. is paid for "gigots," 8d. for other joints, and 7½d. per lb. for sides of mutton; beef is bought in bulk at 6¾d. per lb. for hindquarters and 5¼d. per lb. for forequarters.

At Ayr the prices are 8d. and 10d. per lb.; at Kilmarnock 8d. and 6d., with "hough" 3d. per lb.

At the City of Dublin Hospital the all-round price is 7½d., and in Belfast 7d.

The unhappy student of hospital statistics is forced to the reluctant conclusion that the amount spent in institutions on any article of common consumption rises in direct proportion as the price of the article falls. Although the above comparison of prices shows that in many London hospitals an average of 2d. per pound less is paid for the main meat supplies than in the provinces, and although the diet sheets show that almost precisely the same amount of meat is supplied to the patient in hospitals all over the kingdom, yet the cost of each patient's meat is clearly at least ¾d. a day more in London. It will be remembered that the allowance of £4 a year for each patient's meat, which in London was found to work out correctly in accordance with ascertained figures, was found on examination to be for provincial hospitals in almost every instance excessive, and even the modified estimate of £3 per head seemed in some instances beyond the mark.

And, again, a still more serious discrepancy was observed in the yearly cost of each member of the staff for butcher's meat, obtained by deducting the cost of the patients, and dividing the rest of the butcher's bill among the remaining inmates.

The yearly cost of meat in Scotch hospitals for each member of the staff varies from £8 12s. at the Edinburgh Royal Infirmary to £6 9s. at Aberdeen, the average among five hospitals being £7 7s.

In English provincial hospitals the average yearly butcher's bill of each official works out at from £4 14s. at the Sussex County Hospital to £12 13s. at Sheffield, the average cost among 14 hospitals being £8 per head.

In metropolitan hospitals the range is from £6 per head at the Royal Free to £24 15s. at the London, and the average yearly butcher's bill of each official among 13 hospitals is £11 17s.

It should be borne in mind, in attempting to gauge the full significance of these discrepancies, that an extra penny a day on each patient involves an expenditure of £152 a year for every 100 occupied beds. The difference in expenditure on meat for each member of the staff, between the highest and lowest averages in metropolitan hospitals, is not less than £18, or a yearly difference for every hundred officials of £1,800. And this for one article of diet only.

After the most careful inquiry we have entirely failed to establish the conclusion which seemed at first inevitable, viz., that the standard of living both for patients and staff was appreciably higher in metropolitan than in provincial hospitals. We must, therefore, commend the solution of the mystery to the vigilant attention of those responsible for the administration of these intricate affairs, in the earnest hope that the time is not far distant when public confidence will be invited by a new policy of entire openness and simplicity in hospital management.

